# Is optimal first-line chemotherapy deliverable in all newly diagnosed ovarian cancers? A population-based study

**DOI:** 10.1038/sj.bjc.6601071

**Published:** 2003-09-09

**Authors:** O M McNally, E Delaney, R D Petty, M E Cruickshank, A W Hutcheon, D E Parkin

**Affiliations:** 1 Department of Gynaecology/Oncology, Ward 43, Aberdeen Royal Infirmary, Foresterhill, Aberdeen AB15 2ZN, Scotland; 2 Department of Clinical Oncology, Ward 43, Aberdeen Royal Infirmary, Foresterhill, Aberdeen AB15 2ZN, Scotland

**Keywords:** NICE, ovarian cancer, chemotherapy

## Abstract

NICE guidance recommends the use of paclitaxel and a platinum therapy for all cases of ovarian cancer. We report our experience of treating 133 patients with ovarian cancer over a 3-year period. Where indicated, 91% received chemotherapy. A taxane/platinum combination was found to be appropriate in 63% of patients only.

Since its inception in 1999, the role of the National Institute for Clinical Excellence (NICE) has been to provide patients, healthcare professionals and the public with guidance on best practice, based on available evidence. Guidance on specific health technologies, such as chemotherapy, as well as clinical management of specific conditions are included in this remit. To date, much has been written of the potential impact both financially and on clinical practice of this government ‘watchdog’, but little has been published on whether or not it has been possible for this guidance to be successfully implemented within the National Health Service (NHS). NICE has recommended that ‘paclitaxel in combination with a platinum therapy (cisplatin or carboplatin) should be the standard initial therapy for patients with ovarian cancer following surgery’ (NICE, 2000). The additional cost (outside therapeutic trials and private prescription) to the NHS of using taxanes in patients with ovarian cancer is estimated at 7 million pounds (Dent and Sadler, 2002).

All new cases of ovarian cancer diagnosed in the Grampian region and confirmed by a single pathology laboratory are entered into the gynaecological cancer database at Aberdeen Royal Infirmary. Standard management of ovarian cancer by the designated gynaecological oncology service, outwith ongoing clinical trials, is surgical staging where possible followed by six cycles of first-line combination chemotherapy with carboplatin (AUC6) and paclitaxel (175 mg m^−2^).

The aim of this study was to see if NICE guidance on chemotherapy for ovarian cancer was implementable in our population. We have therefore performed a prospective population-based study to see if adjuvant treatment with carboplatin/cisplatin and a taxane, paclitaxel/docetaxel is deliverable in every newly diagnosed case of ovarian cancer.

## MATERIALS AND METHODS

All patients with newly diagnosed epithelial ovarian cancer entered into the gynaecological database from 1 March 1998 to 1 March 2001 were prospectively followed. For each patient a large number of parameters are entered into the cancer database, but for the purposes of this study attention was confined to the following: age, performance status based on Eastern Cooperative Oncology Group (ECOG), surgical treatment, if any, and adjuvant treatment, that is chemotherapy/radiotherapy. With regard to chemotherapy, the type, dose, duration and indications for withholding or discontinuing treatment were noted.

## RESULTS

During the 36-month period studied, 133 newly diagnosed cases of ovarian cancer were entered into the gynaecological cancer database. A total of 113 patients (85%) had surgical staging. In all, 15 patients had early disease, stage 1, and therefore adjuvant chemotherapy was not indicated. One patient had a mixed Mullerian tumour and a doxorubicin/ifosphamide regime was more appropriately administered. The remaining 117 patients identified as requiring chemotherapy had a pathological diagnosis of epithelial ovarian carcinoma. In all, 34 (29%) of these patients received chemotherapy within a clinical trial. Table 1

**Table 1:** Type of treatment used

**Type of treatment**	***N***	**%**
Carboplatin/paclitaxel	51	43.6
Carboplatin/docetaxel	17	14.5
Carboplatin only	37	31.6
Chlorambucil	2	1.7
Chemo/radiotherapy	1	0.9
Radiotherapy	2	1.7
No treatment	7	6.0
		
Total	117	

outlines the treatment used. One patient declined treatment on social grounds and died within 1 week of diagnosis of disease. Six patients died of disease within 1 month of diagnosis and before treatment could have been commenced. Disease was confined to the pelvis in two patients and the option of adjuvant radiotherapy alone was used. Performance status precluded intravenous chemotherapy in two patients but both received oral chlorambucil. No patient was refused treatment for social reasons.

A platinum/taxane combination was used in 68 of the remaining patients, 64% (*N*=*106*). Docetaxel was administered within the confines of ongoing trials (dose: 100 mg m^−2^ within SCOTROC 1 and 100/75/25 mg m^−2^ within SCOTROC 11). The median age of this group of patients was 58 years (range, 30–83 years). All but one patient had had a laparotomy as part of their management prior to chemotherapy. All but five patients completed six cycles of combination therapy. Two patients had an allergic reaction to the first dose of paclitaxel (2.9%); two patients developed symptoms of neurotoxicity necessitating discontinuing paclitaxel after five cycles and a rising Ca125 in one patient during docetaxel treatment within SCOTROC 11 prompted a change to carboplatin treatment alone. A platinum/taxane combination was not used in the remaining 38 patients. One patient received carboplatin and pelvic irradiation and Table 2

**Table 2:** Main reasons why NICE guidance on chemotherapy was not recommended

**Reason**	***N***	**%**
Age (years)	12	32.4
Performance status (ECOG)	9	24.3
Medical comorbidity	13	35.1
Postoperative complications	3	8.1
Patient choice for milder toxicity	0	0

ECOG=Easterrn Cooperative Oncology Group

lists the reasons why 37 patients received carboplatin only. The median age of these patients was 76 years (range, 50–94 years). In all, 14 patients in this group did not have a laparotomy prior to receiving chemotherapy. All but 13 patients completed six cycles of treatment in whom the indications for cessation or a change of treatment were: a rising Ca125, 6; death, 3; renal impairment, 2; and marrow suppression, 2.

## DISCUSSION

The inequality of the ‘postcode’ delivery system of healthcare seen across the NHS was one of the main reasons why NICE was conceived. The recommendations of NICE should therefore have a significant impact on the quality and consistency of care of patients. The Department of Health expects the recommendations of NICE to be followed and has emphasised this particularly in relation to new drugs for cancer. Such dogma in reality, however, fails to consider other factors such as competing treatments and the particular needs of patients within a given Health Authority at any one time. For the patient and relative, to whom the work of NICE is freely available, NICE may provoke some concern regarding the receipt of appropriate treatment. However, they must be reminded that all NICE guidance documents begin with a caveat that allows clinical judgement to be exercised in any individual case.

The Grampian supra regional service described here is not subject to differences in referral practice or postcode prescribing as might be experienced in other UK regions, particularly where public/private prescribing may have an impact on ultimate treatment. The fact that all cases are centrally referred, and funding for chemotherapy, in particular taxanes, is available irrespective of recruitment to clinical trials provides an ideal group for studying the impact of national guidelines.

In the present study, of those patients in whom it was indicated, all but seven received some form of adjuvant treatment (94%, *N*=117). Overall therefore, age and comorbidity did not preclude treatment, although these two factors constituted the main reasons as to why combination therapy to include a taxane was not recommended in one-third of patients (33%, *N*=117). Sundararajan *et al* (2002) have previously shown a significant variation in the use of chemotherapy in the elderly with patients over the age of 65 years not only less likely to receive any form of chemotherapy but also less likely to receive combination therapy.

Patients with newly diagnosed ovarian cancer represent a heterogeneous group. We report our experience with adjuvant chemotherapy in these patients as a guide to the likelihood of being able to implement the recommendations of NICE for which there is little published data thus far. In view of the recently published ICON3 study, which concludes that single-agent carboplatin or cisplatin are as effective as a paclitaxel/carboplatin combination in survival terms and with a considerably better toxicity profile, a review of the evidence on the use of taxanes in the treatment of ovarian cancer by NICE would be anticipated (ICON Group, 2002). In the meantime, we conclude that even in the presence of an aggressive chemotherapy practice, combination treatment based on present NICE guidance is not deliverable in all patients with ovarian cancer.
